# Personalized management of dyslipidemias in patients with diabetes—it is time for a new approach (2022)

**DOI:** 10.1186/s12933-022-01684-5

**Published:** 2022-11-28

**Authors:** Maciej Banach, Stanisław Surma, Zeljko Reiner, Niki Katsiki, Peter E. Penson, Zlatko Fras, Amirhossein Sahebkar, Francesco Paneni, Manfredi Rizzo, John Kastelein

**Affiliations:** 1 Department of Preventive Cardiology and Lipidology, Medical University of Lodz (MUL), Rzgowska 281/289, 93-338 Lodz, Poland; 2 Department of Cardiology and Congenital Heart Diseases of Adults, Polish Mother’s Memorial Hospital Research Institute (PMMHRI), Lodz, Poland; 3 Cardiovascular Research Centre, University of Zielona Gora, Zielona Gora, Poland; 4 Faculty of Medical Sciences in Katowice, Medical University of Silesia in Katowice, Katowice, Poland; 5 Club of Young Hypertensiologists, Polish Society of Hypertension, Gdansk, Poland; 6 Department of Internal Diseases, University Hospital Center Zagreb School of Medicine, Zagreb University, Zagreb, Croatia; 7 Department of Nutritional Sciences and Dietetics, International Hellenic University, Thessaloniki, Greece; 8 School of Medicine, European University of Cyprus, Nicosia, Cyprus; 9 Clinical Pharmacy and Therapeutics Research Group, School of Pharmacy and Biomolecular Sciences, Liverpool John Moores University, Liverpool, UK; 10 Liverpool Centre for Cardiovascular Science, Liverpool, UK; 11 Department of Vascular Disease, University Medical Center Ljubljana, Ljubljana, Slovenia; 12 Faculty of Medicine, University of Ljubljana, Ljubljana, Slovenia; 13 Biotechnology Research Center, Pharmaceutical Technology Institute, Mashhad University of Medical Sciences, Mashhad, Iran; 14 Applied Biomedical Research Center, Mashhad University of Medical Sciences, Mashhad, Iran; 15 University Heart Center, Department of Cardiology, University Hospital Zurich, Zurich, Switzerland; 16 Center for Translational and Experimental Cardiology (CTEC), University Hospital Zurich and University of Zurich, Zurich, Switzerland; 17 Promise Department, School of Medicine, University of Palermo, Palermo, Italy; 18 College of Medicine, Mohammed Bin Rashid University of Medicine and Health Sciences, Dubai, UAE; 19 Department of Vascular Medicine, Academic Medical Center, University of Amsterdam, Amsterdam, The Netherlands

**Keywords:** Cardiovascular risk, Diabetes, Individual therapy approach, Lipid lowering therapy, Statins

## Abstract

Dyslipidemia in patients with type 2 diabetes (DMT2) is one of the worst controlled worldwide, with only about 1/4 of patients being on the low-density lipoprotein cholesterol (LDL-C) target. There are many reasons of this, including physicians’ inertia, including diabetologists and cardiologists, therapy nonadherence, but also underusage and underdosing of lipid lowering drugs due to unsuitable cardiovascular (CV) risk stratification. In the last several years there is a big debate on the risk stratification of DMT2 patients, with the strong indications that all patients with diabetes should be at least at high cardiovascular disease (CVD) risk. Moreover, we have finally lipid lowering drugs, that not only allow for the effective reduction of LDL-C and do not increase the risk of new onset diabetes (NOD), and/or glucose impairment; in the opposite, some of them might effectively improve glucose control. One of the most interesting is pitavastatin, which is now available in Europe, with the best metabolic profile within statins (no risk of NOD, improvement of fasting blood glucose, HOMA-IR, HbA1c), bempedoic acid (with the potential for the reduction of NOD risk), innovative therapies—PCSK9 inhibitors and inclisiran with no DMT2 risk increase, and new forthcoming therapies, including apabetalone and obicetrapib—for the latter one with the possibility of even decreasing the number of patients diagnosed with prediabetes and DMT2. Altogether, nowadays we have possibility to individualize lipid lowering therapy in DMT2 patients and increase the number of patients on LDL-C goal without any risk of new onset diabetes and/or diabetes control worsening, and in consequence to reduce the risk of CVD complications due to progression of atherosclerosis in this patients’ group.

## Introduction

Type 2 diabetes (DMT2) has been recognized as one of the major risk factors for cardiovascular diseases (CVDs). In 1990, the worldwide prevalence of diabetes was 211.2 million individuals, and in 2021 patients with a diagnosis of DMT2 increased to 536.6 million, while it is projected to reach 783.2 million people globally in 2045 [1, 2]. Thus, type II diabetes is an ever-increasing issue of epic proportions in modern medicine.

### Patients with diabetes should be classified as having high CV risk

Adults with diabetes have a four times higher atherosclerotic cardiovascular disease (ASCVD) risk compared with non-diabetic adults, and this cardiovascular (CV) risk rises with worsening glycaemic control. Diabetes has been associated with a 75% increase in all-cause mortality in adults, and CVD accounts for a large part of the excess mortality [3]. Despite intense debate, the recent recommendations of the European Society of Cardiology (ESC) 2021 on prevention indicated that DMT2 patients may be classified as being at moderate CV risk (patients with well controlled diabetes of recent onset [e.g. < 10 years], no evidence of target organ damage [TOD] and no additional ASCVD risk factors) [4]. However, we may very seldom observe such patients in clinical practice—as almost all our DMT2 patients exhibit other risk factors and/or subclinical organ damage, when carefully assessed. Thus, for this group the ASCVD risk is often underestimated and consequently undertreated [5]. Moreover, this ASCVD risk increases sharply many years before the diabetes is diagnosed.

At the time of diagnosis, macro- and microvascular changes are already present in a significant proportion of patients, as Gerstein et al*.* reported in 1997 [6]. A subsequent meta-analysis by Cai et al. showed that prediabetes was associated with an increased risk of all-cause mortality (relative risk [RR] = 1.13, 95% CI: 1.10–1.17), CVDs (RR = 1.15, 95% CI: 1.11–1.18), coronary heart disease (RR = 1.16; 95% CI: 1.11–1.21), and stroke (RR = 1.14; 95% CI: 1.08–1.20) after a median follow-up time of 9.8 years [7]. Moreover, the meta-analysis of Gujral et al. which included 1.85 million subjects, showed that impaired glucose tolerance and elevated fasting glucose significantly increased the risk of macrovascular and microvascular complications [8]. De Simone et al*.* also reported that end-organ damage such as left ventricular hypertrophy, left atrial dilatation and high urinary albumin/creatinine ratio occurred in large proportion of patients before the clinical diagnosis of type 2 diabetes. [9]

**Fig. 1:**
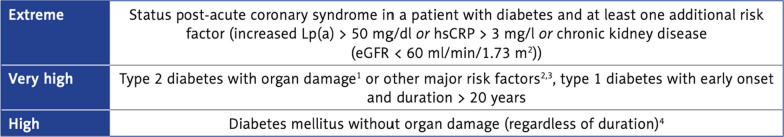
Cardiovascular risk categories in patients with diabetes mellitus according to the Polish Lipid Association 2021 (with permission). [1, 13] ^1^Target organ damage is defined as the presence of microalbuminuria, retinopathy, neuropathy, and/or left ventricular myocardial damage; ^2^“Other” means 2 or more; ^3^Major risk factors include: age ≥ 65 years, hypertension, dyslipidaemia, smoking, obesity; ^4^Not applicable to young adults (< 35 years of age) with type 1 diabetes lasting < 10 years. *Lp(a)* lipoprotein(a), *hsCRP* high-sensitivity C-reactive protein, *eGFR* estimated glomerular filtration rate

Moreover, at the DMT2 diagnosis, other CV risk factors typically coexist, such as abdominal obesity, dyslipidemia, arterial hypertension, and metabolic sydnrome [10, 11]. The 2021 ESC prevention guidelines [4] are too complex for practitioners to stratify the risk of diabetic patients and this may result in inappropriate treatment of some patients [12]. Taking this into account, the authors strongly suggest a change in the approach to risk stratification among diabetic patients e.g. according to the guidelines of the Polish Lipid Association (PoLA) and 5 other major Polish scientific societies from 2021. Hence, in the PoLA guidelines, patients with diabetes are immediately classified as having either high, very high or extreme CV risk (Fig. 1) [13].

### Goals and rules of lipid-lowering treatment in patients with diabetes and dyslipidemia

**Fig. 2:**
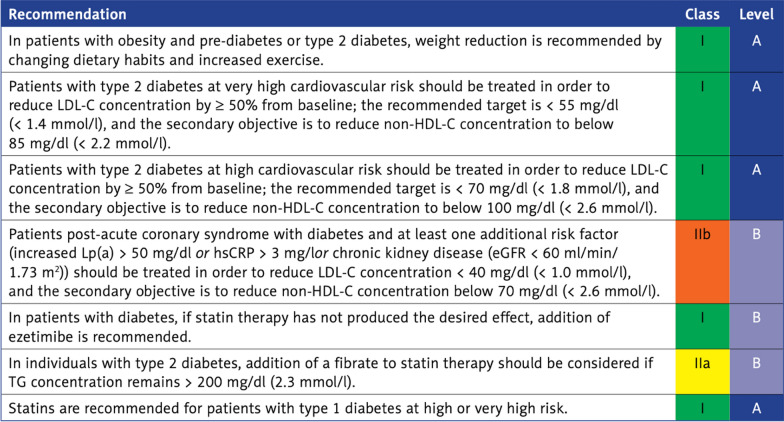
Recommendations by Polish Lipid Association 2021 on treatment of lipid disorders in patients with diabetes (with permission). [13] *LDL-C* low-density lipoprotein cholesterol, *HDL-C* high-density lipoprotein cholesterol, *Lp(a)* lipoprotein a, *hsCRP* high-sensitivity C-reactive protein, *eGFR* estimated glomerular filtration rate

More than 1/3 of diabetic patients have atherogenic dyslipidemia, which further increases the risk of atherosclerotic CVD [14]. As shown in the AHEAD (Action for Health in Diabetes) study, the presence of dyslipidemia in patients with type 2 diabetes significantly increased the risk of ASCVD (hazard ratio [HR] = 1.30; 95% CI: 1.03–1.63) and coronary artery disease (HR = 1.48; 95% CI: 1.14–1.93) [15]. In patients with diabetes and coexistence of lipid disorders, it is recommended to reduce low density lipoprotein cholesterol (LDL-C) levels to < 1.8 mmol/l (< 70 mg/dl) and to achieve at least a reduction of 50% of the baseline value (Fig. 2) [13].

In the case of lipid disorders in diabetic patients, practitioners should follow the principle: *"the lower, the better",* but also to strive for *“the earlier, the better”* as soon as possible and maintain it as long as possible, preferably throughout the patient’s lifetime *“the longer, the better”*. Such an approach will significantly reduce the risk of CVD in these patients [13]. The paradigm of “the lower, the better” has been confirmed in hundreds of trials and meta-analyses, therefore we would like to explain the importance of the two additional rules.

#### Paradigm II: the earlier, the better

Increased LDL-C is associated with a significant increase in the risk of ASCVD. A 16% (HR = 1.16; 95% CI: 1.12–1.21) increase in risk is observed for every additional 1 mmol/l of LDL-C. Among subject aged 20–49 this relationship is much stronger, and here a 47% (HR = 1.47; 95% CI: 1.32–1.64) increase in risk is observed for every additional 1 mmol/l of LDL-C [16]. A study by Navar-Boggan et al*.* showed that the incidence of moderate dyslipidemia in young adults who were not treated with statins increased the risk of coronary artery disease by 67% (HR = 1.67; 95% CI: 1.06–2.64) over 15 years of follow-up [17]. The atherogenic effect of LDL-C appears to be dependent on both the concentration of circulating LDL-C and the duration of exposure to this concentration [18]. Therefore, it is especially important for very high and extremely high risk patients to achieve LDL-C targets through the use of intensive lipid lowering therapy to significantly reduce atheroma plaque volume and to stabilize plaques. For many patients, this will require immediate (upfront) combination therapy, as the extent of lipid-lowering required to reach targets would not be achievable with monotherapy [13, 19–21]

#### Paradigm III: the longer, the better

The consequences of statin discontinuation on the risk of major CV events were assessed by Thompson et al*.* in a study involving 67,418 older, long-term statin users. It was shown that patients who discontinued statin therapy exhibited an approximately 30% higher risk of major adverse CV events during the 6-year follow-up [22]. In a study by Rannanheimo et al*.* covering 97,575 new statin users aged 45 to 75 years followed for 3 years, it was shown that those with better adherence had a significantly better prognosis (25% lower risk of any CV event or death) than those with low-adherence. Good adherers also had a lower incidence of acute coronary syndrome (ACS) (HR = 0.56; 95% CI: 0.49–0.65) and acute cerebrovascular events (HR = 0.67; 95% CI: 0.60–0.76) compared to poor adherers [23]. A meta-analysis by Martin-Ruiz et al*.* found that patients with best adherence to statin therapy showed a significant reduction in risk of ischemic heart disease (IHD) by 18%, CVD by 47%, cerebrovascular disease by 26% and death by 49% compared to patients with the worst adherence [24]. In the study by Giral et al*.* it was found that stopping statin use led to a significant increase in the risk of any CV event (HR = 1.33; 95% CI: 1.18–1.50), coronary event (HR = 1.46; 95% CI: 1.21- 1.75), and cerebrovascular event (HR = 1.26; 95% CI: 1.05–1.51) [25]. Finally, Ference et al*.* showed that those adherent patients being on LDL-C target for at least 5 years may expect at least a 25% ASCVD risk reduction, and those having lifetime adherence (40 years or longer) even a 55% ASCVD risk reduction [26].

### Optimization of lipid-lowering treatment in patients with diabetes and dyslipidemia

**Fig. 3:**
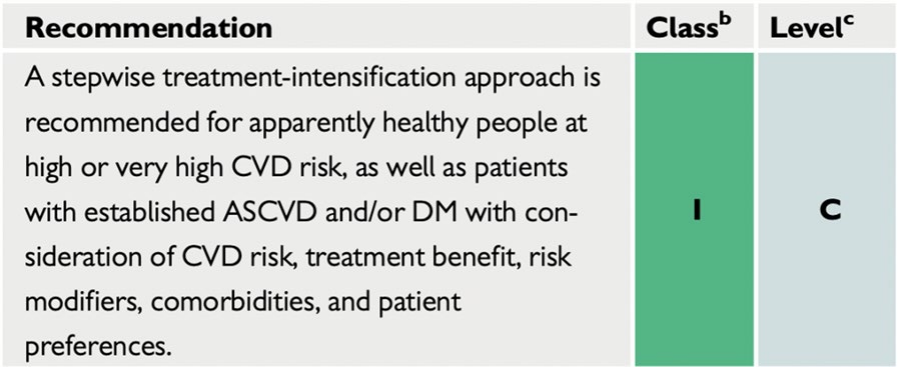
2021 European Society of Cardiology recommendation on low-density lipoprotein cholesterol goals. [4] *CVD* cardiovascular risk, *ASCVD* atherosclerotic cardiovascular disease, *DM* diabetes mellitus

In our opinion, the stepwise approach to intensification of lipid-lowering treatment in patients with diabetes (as recommended by ESC) (Fig. 3) [4] should be replaced with a one-step intensive lipid lowering treatment strategy to follow the abovementioned paradigms and to really prevent first and recurring CVD events.

Moreover, with the advent of novel therapies and those expected to be available soon, we now have the potential to offer effective individualized treatment of dyslipidemia in patients with diabetes. The main rules to remember to be effective with lipid lowering therapy (LLT) are: (1) correctly assess the ASCVD risk of the diabetic patient; (2) determine the baseline LDL-C level to be able to calculate the expected absolute LDL-C reduction needed for your patient to reach their target; (3) explore the full lipid and lipoprotein profile to tailor the therapy to the specific dyslipidemia.

#### Pitavastatin

Pitavastatin, which is finally available on European market, seems to be an excellent choice for patients at high risk of diabetes (the elderly, with metabolic disorders, obesity, metabolic syndrome, insulin resistance in the course of various diseases), pre-diabetes and finally diabetes with concomitant metabolic disorders [13, 27]. Pitavastatin is the third most potent statin. At a dose of 4 mg, daily it reduces LDL-C by approximately 44% (range: 43–47%) [28]. A meta-analysis by Seo et al*.* that included 10,238 new pitavastatin users (15,998 person-years of follow-up) and 18,605 atorvastatin and rosuvastatin users showed that pitavastatin use was associated with a significantly lower risk of new-onset diabetes (NOD) in comparison to the atorvastatin and rosuvastatin (HR = 0.72; 95% CI: 0.59–0.87) [29]. It is also worth emphasizing that administration of highest-dose pitavastatin (4 mg) did not increase the risk of NOD in patients at high risk of developing diabetes during 3-years of follow-up [30]. Pitavastatin has also been shown to reduce HbA_1C_ levels in patients with poorly controlled type 2 diabetes [31]. Moreover, it is the most effective statin for improving the atherogenic profile of patients with diabetes, and has the most potent high density lipoprotein cholesterol (HDL-C)-raising effect (increases above 20% may occur) [32], and significantly reduces triglycerides (TG) (− 18–25%) [33, 34]. An randomized controlled trial by Moroi et al. which included 664 patients, including 76% with diabetes, showed that pitavastatin therapy compared with atorvastatin may more effectively prevent CV events in hypercholesterolemic patients with one or more risk factors for ASCVD despite having similar effects on LDL-C concentration [35].

Pitavastatin monotherapy could be recommended for patients with diabetes who are required to reduce their LDL-C by 40 to 50% from baseline.

#### Ezetimibe

When the target LDL-C reduction exceeds 50%, the combination of pitavastatin, or rosuva- and atorvastatin, bearing in mind that these latter drugs increase the NOD risk, with ezetimibe might be considered [36]. In the randomized HIJ-PROPER study involving 1734 ACS patients with dyslipidemia, it was shown that the addition of ezetimibe to pitavastatin resulted in an additional reduction of LDL-C by 14%, enabling a 52% reduction in LDL-C which was associated with a significant 23% reduction of event rate and other outcomes—with 23% reduction of stroke risk and 55% reduction of all-cause mortality [37]. The addition of ezetimibe to statin therapy is associated with greater efficacy than statin monotherapy in reducing the incidence of CVD and stroke among diabetic patients (RR = 0.69; 95% CI: 0.67–0.73 and RR = 0.74; 95% CI: 0.56–0.98, respectively), as shown in the meta-analysis of 136,893 subjects carried out by Miao et al*.* [38]. It was also confirmed in the recent randomised, open-label, non-inferiority trial on the long-term efficacy and safety of moderate-intensity statin with ezetimibe combination therapy versus high-intensity statin monotherapy in ASCVD patients (the RAndomized Comparison of Efficacy and Safety of Lipid-lowerING With Statin Monotherapy Versus Statin/Ezetimibe Combination for High-risk Cardiovascular Diseases [RACING] study) (37% patients with DMT2). The authors showed that moderate-intensity statin with ezetimibe combination therapy was non-inferior to high-intensity statin monotherapy for the 3-year composite outcomes with a higher proportion of patients with LDL-C concentrations of < 70 mg/dL and lower intolerance-related drug discontinuation or dose reduction [39]. In a meta-analysis of 48 RCTs by Wang et al*.* ezetimibe did not influence the risk of NOD (RR = 0.88; 95% CI: 0.61–1.28) [39]. Despite inconsistent data, it is worth mentioning, that ezetimibe may reverse insulin resistance, reduce lipid dysmetabolism after a meal and improve endothelial function in patients with metabolic syndrome and coronary artery disease [40].

The addition of ezetimibe to statin therapy (preferably as a fixed-dose combination [FDC]) should be recommended for patients with diabetes who are required a reduction of LDL-C by 50–65% from baseline.

#### Bempedoic acid

In the clinical scenario of patients with diabetes where the required reduction of LDL-C exceeds 60%, the use of a triple therapy may be beneficial: statin plus ezetimibe plus bempedoic acid. Bempedoic acid reduces LDL-C by up to 25% and in combination with ezetimibe by as much as 40% [41–43]. Triple therapy, including a moderate dose of statin is associated with up to 70% LDL-C reduction [44]. In a study by Leiter et al. which included 3621 subjects who were randomized 2:1 to monotherapy oral bempedoic acid 180 mg or placebo once daily for 12 to 52 weeks, it was shown that bempedoic acid significantly lowered LDL-C (up to approximately 30%) and HbA_1C_ by -0.12% and did not worsen fasting glucose *vs.* placebo. Moreover, bempedoic acid was found to not increase the incidence of NOD *vs.* placebo over a median follow-up of 1 year [45]. A meta-analysis of 11 studies by Wang et al*.* showed that bempedoic acid reduced the risk of CV events by 25% (RR = 0.75; 95% CI: 0.56–0.99) and the risk of NOD by 35% in patients with hypercholesterolaemia (RR = 0.65; 95% CI: 0.44–0.96) [46]. An even greater reduction of NOD (41%) was observed in the meta-analysis of 10 RCTs with 3788 patients comprising 26 arms (active arm [n = 2460]; control arm [n = 1328]) by Cicero et al. [47]. Based on the data from phase 3 trials adverse events (AEs) related to bempedoic acid lead to slightly higher discontinuation in comparison to placebo (13.4/100 and 8.9/100 person-years, respectively). Bempedoic acid might be also associated with mild increases (clinically irrelevant) in creatinine (by mean 0.048 mg/dL), and uric acid (by mean 0.82 mg/dL) and mild reversible decreases in hemoglobin (reductions of ≥ 2 g/dL in 4.9/100 vs 2.0/100 person-years, respectively). Gout incidence was 1.6/100 vs 0.5/100 PY in the bempedoic acid vs placebo groups, especially in those with the previous history of gout or high baseline levels of uric acid [48, 49].

The addition of bempedoic acid plus ezetimibe (preferably as an FDC) to statin therapy should be recommended for patients with diabetes who are required to reduce LDL-C by 65–80% from baseline.

#### PCSK9 inhibitors and inclisiran

When the required reduction of LDL-C exceeds 80%, a proprotein convertase subtilisin/kexin 9 (PCSK9) modulator (alirocumab, evolocumab or inclisiran) should be added to the current LLT.

Alirocumab and evolocumab reduce LDL-C by about 60% in patients with diabetes and dyslipidemia, and also have a very positive effect on other lipid fractions in patients with diabetes: TG (− 15–30%), apolipoprotein B (− 35%) and Lp(a) (− 20–30%) [50, 51]. Next, inclisiran enables the reduction of LDL-C by 50–55%, non-HDL-C by 45%, apolipoprotein B by 41% and TG by 10% [52, 53]. PCSK9 modulators significantly reduce the risk of CV and CV death among patients with CVD (RR = 0.80; 95% CI: 0.73–0.87) and this effect might be even greater in those with pre- and diabetes [54–56]. Therapies with alirocumab, evolocumab, and inclisiran were not associated with an increased incidence of NOD [57]. The recently published FOURIER-OLE (Further Cardiovascular Outcomes Research With PCSK9 Inhibition in Subjects With Elevated Risk-Open-Label Extension) study with 6635 patients (with about 34% patients with diabetes) and the follow-up of over 8 years confirmed both paradigms—the earlier on LDL-C goal, the better but also the longer the better, as evolocumab led to further reductions in cardiovascular events compared with delayed treatment initiation (15% lower risk of CV death, myocardial infarction, stroke, or hospitalization for unstable angina or coronary revascularization, 20% lower risk of CV death, myocardial infarction, or stroke, and 23% lower risk of CV death. Incidences of AEs, including new-onset diabetes did not increase over time [58].

The addition of a PCSK9 targeted approach therapy to statin plus ezetimibe (preferably as an FDC) should be recommended in patients with diabetes who are required to reduce LDL-C by more than 80% from baseline.

### Effective reduction of triglyceride rich lipoproteins

In patients who still have an elevated TG concentration (and/or non-HDL-C, which is now an equivalent goal to LDL-C), despite intensive LDL-C lowering treatment (using the drugs described above), the use of fenofibrate (in diabetic patients without ASCVD to reduce the risk of micro- and macrovascular complications) or icosapent-ethyl (in diabetic patients with ASCVD) is recommended [13, 59]. The ACCORDION study showed that the addition of fenofibrate to a statin in T2DM patients and dyslipidemia (baseline TG: 327.2 ± 125.3 mg/dl) led to a reduction in the risk of all-cause death in the long term—9.7 years follow-up from time of randomization—(HR: 0.68; 95% CI: 0.52–0.88), death from CVD (0.63; 95% CI: 0.42–0.95) and severe coronary heart disease (0.66; 95% CI: 0.51–0.86) [60]. The beneficial effect of fenofibrate on the prognosis of DMT2 patients and dyslipidemia was also confirmed by Koopal et al*.* In a study involving 17,142 patients from the FIELD (the Fenofibrate Intervention and Event Lowering in Diabetes), ACCORD (the Action to Control Cardiovascular Risk in Diabetes), and SMART (Second Manifestations of ARTerial disease) studies [61]. Unfortunately, the Pemafibrate to Reduce Cardiovascular OutcoMes by Reducing Triglycerides IN patiENts With diabeTes (PROMINENT) phase 3 study with pemafibrate was prematurely discontinued (April 2022) due to the unlikely achievement of the primary endpoint [62–64]. The REDUCE-IT (Reduction of Cardiovascular Events With Icosapent Ethyl–Intervention Trial) showed that the use of icosapent ethyl (IPE) in patients with high TG and diabetes type 2 led to a reduction risk of CV death, nonfatal myocardial infarction, nonfatal stroke, coronary revascularization, or unstable angina by 25% (HR = 0.77; 95% CI: 0.68–0.87). It should be emphasized, however, that IPE increased the risk of atrial fibrillation/flutter: 3.1 vs. 2.1%, P = 0.004) [65]. In addition, based on the JELIS study, it is recommended to use EPA at a dose of 1.8 g/day (and more), because it was shown that such therapy reduced the risk of major CV events in secondary prevention by 19% (HR = 0.81; 95% CI: 0.657–0.998) [66]. Based on the Polish guidelines, general recommendations for omega-3 acids applications were suggested as following: “In at least high-risk patients with TG ≥ 2.3 mmol/l (≥ 200 mg/dl) despite statin therapy, omega-3 acids (polyunsaturated fatty acids [PUFA] in a dose of 2 to 4 g/day) in combination with a statin may be considered (IIbC) [13, 67].

**Fig. 4:**
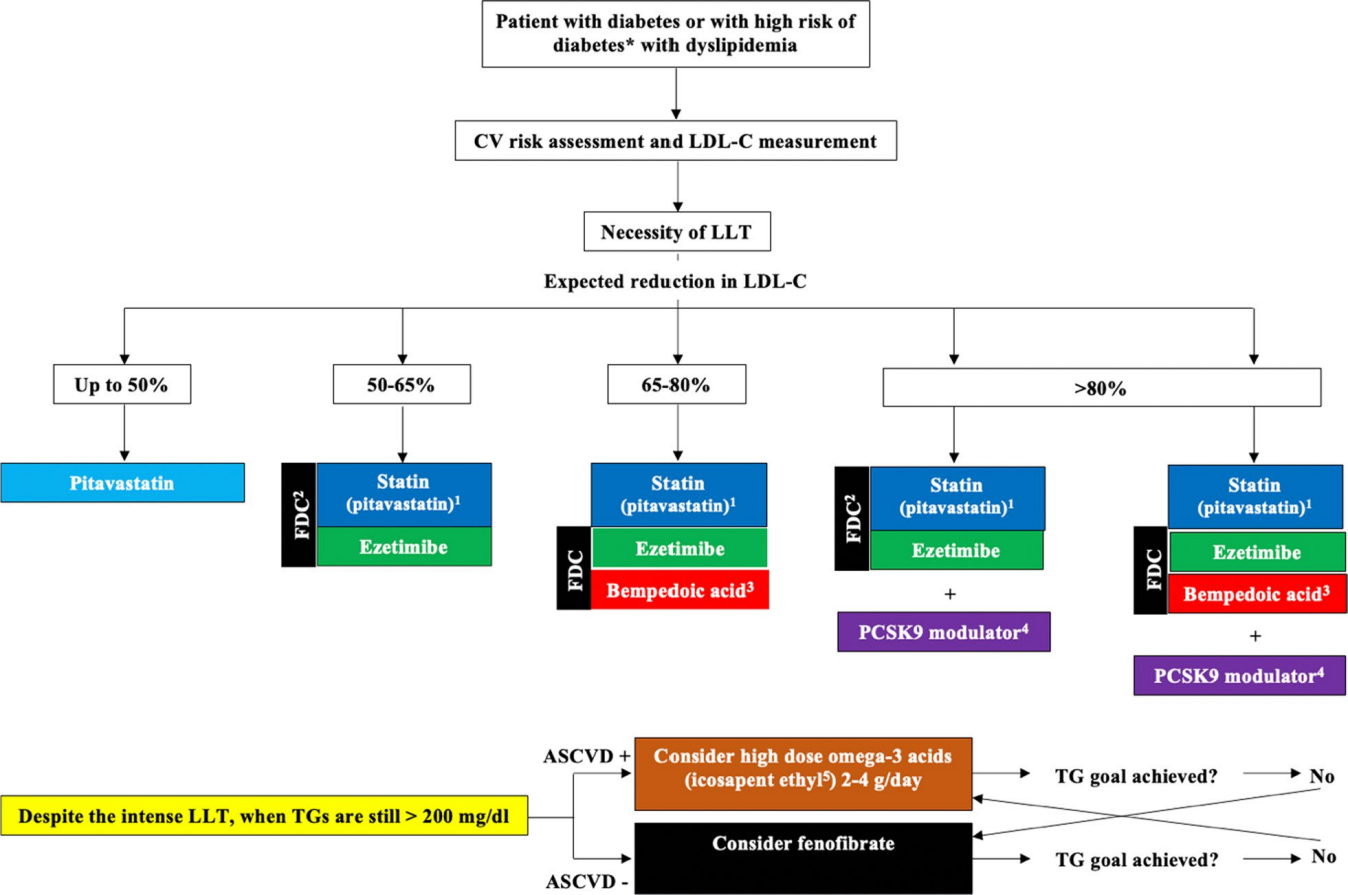
Proposed therapy scheme for patients with diabetes and dyslipidemia. *CV* cardiovascular, *LDL-C* low density lipoprotein cholesterol, *LLT* lipid lowering therapy, *FDC* fixed dose combination, *PCSK9* proprotein convertase subtilisin/kexin 9, *TG* triglyceride, *ASCVD* atherosclerotic cardiovascular disease. LLT in maximum, tolerated doses. *Patients at the risk of diabetes (with metabolic disorders, obesity, metabolic syndrome, insulin resistance in the course of various diseases), pre-diabetes and finally diabetes with concomitant metabolic disorders^. 1^Pitavastatin is preferable; ^2^In case of other statin—rosuvastatin or atorvastatin—always consider fixed dose combination with ezetimibe; ^3^FDC of statin and ezetimibe plus BA might also be an option; ^4^In most of the countries, reimbursement criteria does not allow the upfront triple combination therapy with PCSK9 inhibitors; Inclisiran is still not reimbursed in many countries; ^5^IPE is still not available in Europe

The personalized recommendations of management in patients with diabetes and dyslipidemia to reduce the risk associated with atherogenic dyslipidemia and improve (do not worsen) glucometabolic status is presented in Fig. 4.

## Conclusions

The above recommendations aim to optimize LLT in high-risk patients with diabetes (which should also refer to those with prediabetes/metabolic disturbances), where ASCVD risk is often underestimated and undertreated. In fact, fewer than 30% of type II diabetes patients reach their LDL-C targets, irrespective of CVD risk. Due to the concise and practical form of this guidance paper, we have decided to mainly focus on the largest unmet need in diabetic patients—LDL-C goal achievement to clearly show that with the currently available LLTs and suitable approach we can easily be on the LDL-C target in this group of patients at high CVD risk. Thus, we do not discuss the role of non-HDL-C, which is now an equivalent lipid biomarker to LDL-C [13, 67], the remnants—and their important role in CV risk prediction in patients with atherogenic dyslipidemia [68], as well as apolipoprotein B (ApoB)—which has the largest predictive power, but it is still measured definitely too rarely [4, 13, 67]. We are also aware that for some of the above suggested recommendations, additional outcome data is required. It is undoubtedly true for pitavastatin, which has been much less extensively investigated in comparison to rosuvastatin and atorvastatin, for which we still need real-world data. Further evidence is required for the use of early combination therapy, as well for the novel compounds, inclisiran and bempedoic acid. Further investigations and new data are also required for the approach suggested to promote the reduction of TG associated ASCVD residual risk with omega-3 acids. In this context, we are also awaiting new therapies that may further improve the suggested therapeutic paradigms, including evinacumab [69, 70], apabetalone [71], and obicetrapib [72].

Overall, patients with diabetes should be carefully examined in terms of CV risk stratification (e.g., taking into consideration TOD, renal function, subclinical atherosclerosis, etc.), to define their lipid goals. We strongly suggest that, for such high-risk patients, LDL-C target should be achieved as early as possible to maximize CVD prevention.

## Data Availability

Not applicable.

## Abbreviations

ACCORD: The Action to Control Cardiovascular Risk in Diabetes study
ACS: Acute coronary syndrome
AE: Adverse event
AHEAD: The Action for Health in Diabetes study
ApoB: Apolipoprotein B
ASCVD: Atherosclerotic cardiovascular disease
CV: Cardiovascular
CVD: Cardiovascular disease
DMT2: Type 2 diabetes
ESC: European Society of Cardiology
FDC: Fixed-dose combination
FIELD: The Fenofibrate Intervention and Event Lowering in Diabetes trial
HDL-C: High density lipoprotein cholesterol
HR: Hazard ratio
IHD: Ischemic heart disease
LDL-C: Low density lipoprotein cholesterol
LLT: Lipid lowering therapy
NOD: New-onset diabetes
PCSK9: Proprotein convertase subtilisin/kexin 9
PoLA: Polish Lipid Association
PROMINENT: The Pemafibrate to Reduce Cardiovascular OutcoMes by Reducing Triglycerides IN patiENts With diabeTes study
PUFA: Polyunsaturated fatty acids
RACING: The RAndomized Comparison of Efficacy and Safety of Lipid-lowerING With Statin Monotherapy Versus Statin/Ezetimibe Combination for High-risk Cardiovascular Diseases study
REDUCE-IT: The Reduction of Cardiovascular Events With Icosapent Ethyl–Intervention Trial
RR: Relative risk
SMART: Second Manifestations of ARTerial disease study
TG: Triglycerides
TOD: Target organ damage
